# Isolation and identification of a 2,3,7,8-Tetrachlorodibenzo-P-dioxin degrading strain and its biochemical degradation pathway

**DOI:** 10.1007/s40201-021-00626-9

**Published:** 2021-02-24

**Authors:** Lina Qiu, Weiwei Zhang, Aijun Gong, Jiandi Li

**Affiliations:** 1 School of Chemistry and Biological Engineering, University of Science and Technology Beijing, Beijing, 100083 People’s Republic of China; 2 Beijing Key Laboratory for Science and Application of Functional Molecular and Crystalline Materials, University of Science and Technology Beijing, 100083 Beijing, China; 3 Basic Experimental Center for Natural Science, University of Science and Technology Beijing, Beijing, 100083 China

**Keywords:** 2,3,7,8-TCDD, Biodegradation, *Penicillium* sp., Kinetics, Degradation pathway

## Abstract

This study aims to find a high-efficiency degradation strain which can biodegrade the 2,3,7,8-Tetrachlorodibenzo-P-dioxin (2,3,7,8-TCDD). In this paper, a new fungus strain was isolated from activated sludge of Dagu Drainage River in Tianjin which was able to degrade 2,3,7,8-TCDD in the medium. Based on its morphology and phylogenetic analysis of its 18S rDNA sequence, the strain was identified as *Penicillium* sp. QI-1. Response surface methodology using central composite rotatable design of cultural conditions was successfully employed for optimization resulting in 87.9 % degradation of 2,3,7,8-TCDD (1 µg/mL) within 6 days. The optimum condition for degrading 2,3,7,8-TCDD was at 31℃ and pH 7.4. The biodegradation process was fitted to a first-order kinetic model. The kinetic equation was C_t_=0.939e^− 0.133t^ and its half-life was 5.21d. The fungus strain degraded 2,3,7,8-TCDD to form intermediates, they were 4,5-Dichloro-1,2-benzoquinone, 4,5-Dichlorocatechol, 2-Hydrooxy-1,4-benzoquinone, 1,2,4-Trihydroxybenzene and β-ketoadipic acid. A novel degradation pathway for 2,3,7,8-TCDD was proposed based on analysis of these metabolites. The results suggest that *Penicillium* sp. QI-1 may be an ideal microorganism for biodegradation of the 2,3,7,8-TCDD-contaminated environments.

## Introduction

Domestic and industrial activities generate high amounts of wastewater, whose direct disposal to natural channels causes a negative impact on the environment. Dioxins and polychlorinated biphenyls (PCBs), produced as unwanted byproducts of chemical manufacturing, waste incineration, and utility of fossil fuel, are considered to be one of the most hazardous man-made compounds [1, 2]. For example, in the process of producing caustic soda by Chor-alkali method, a large amount of salt sludge is produced, and its composition and discharge are closely related to the content of raw salt impurities as well as the production process. Generally, every 1 ton of caustic soda will produce 40-60kg (dry base) of salt sludge [3, 4]. The concentration of dioxins in electrolytic salt sludge of a Chor-alkali plant was as high as 378.85ug/kg, and its toxic equivalent I-TEQ value was 21.65ug/kg. Most manufacturers, especially small and medium-sized Chor-alkali plants, did not carry out effective treatment. Untreated salt sludge was piled up in and out of the site, or discharged into the rivers, lakes and seas near the plant, causing serious pollution. As a major producer of Chor-alkali, waste residue from Chor-alkali production can be regarded as one of the main sources of dioxins [5].

**Fig. 1:**
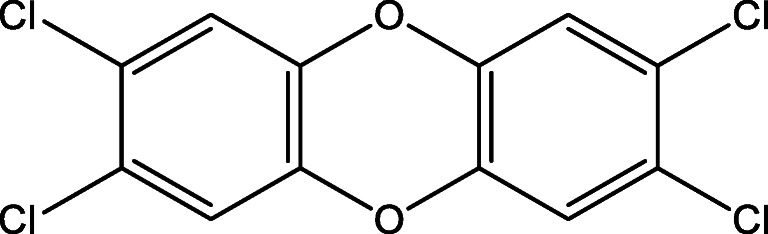
The molecular structure of 2,3,7,8-TCDD

Their distribution in the environment is mainly due to their persistency and bioaccumulation in the food chain. Several studies have reported that 2,3,7,8-Tetrachlorodibenzo-P-dioxin (2,3,7,8-TCDD), which is the most toxic of all PCBs, causes chloracne in humans, skeletal deformities, kidney defects, and weakened immune responses [6]. Figure 1 showed the structure of 2,3,7,8-TCDD. The environmental toxicant 2,3,7,8-TCDD is produced from vehicular exhausts and crematories [7]. It accumulates easily in the environment and most organisms due to its high lipophilicity. Moreover, its resistance to degradation allows it to bioaccumulate in the food chain [8]. TCDD exposure has been implicated in a myriad of adverse health effects in humans. Acute occupational exposure to TCDD after industrial accidents can reportedly induce symptoms like chloracne, porphyria, transient hepatotoxicity, and peripheral and central neurotoxicity [9].

These chemicals are of great health and environmental concern because of their high toxicity, widespread occurrence, and persistence in the environment. They had chemically stable structures and accumulated in the environment over a long time. The average half-life calculated for these congeners was 12 years. The half-life of the most toxic 2,3,7,8-TCDD was approximately ten years [10].

Several remediation technologies have been developed or being developed for treating these chemicals, including incineration and thermal treatment [11], Photolysis [12], photocatalysis [13], c-radiolysis [14], biodegradation [15], degradation by bacteria [16], and dechlorination with zero valent metals [17–22].

Biodegradation studies were initiated in the mid-1980s which demonstrated the microbial conversion of PCDD and PCDF by isolated microorganisms. An early report indicated that lignin-degrading white-rot fungi, as exemplified by Phanerochaete chrysosporium, can degrade an extremely diverse group of environmental pollutants. White-rot fungi can degrade lignin, a complex high-molecular-weight aromatic polymer, as well as a wide spectrum of recalcitrant Organic pollutants, including polycyclic aromatic hydrocarbons (PAHs), polychlorinated biphenyls (PCBs) and polychlorinated phenols. Previous reports have shown that several white-rot fungi belonging to the *Phanerochaete* and *Phlebia genera* degraded PCDDs and PCDFs.

Fungi have been shown to have the ability to degrade a variety of persistent organic pollutants including dioxins. However, only a limited number of studies have focused on fungal degradation of the most toxic dioxin, 2,3,7,8-TCDD mainly due to the strong resistance to biological breakdown of its chemical structure. In this study, we have demonstrated the ability of the fungus *Penicillium* sp. QI-1 to degrade 2,3,7,8-TCDD. The *Penicillium* sp. QI-1 has the ability to decrease dioxin concentration during the 7 days of incubation. The biodegrability, degradation kinetics and biochemical degradation pathway were the first to evaluate.

## Materials and methods

### Material and fungal inoculum preparation

**Fig. 2:**
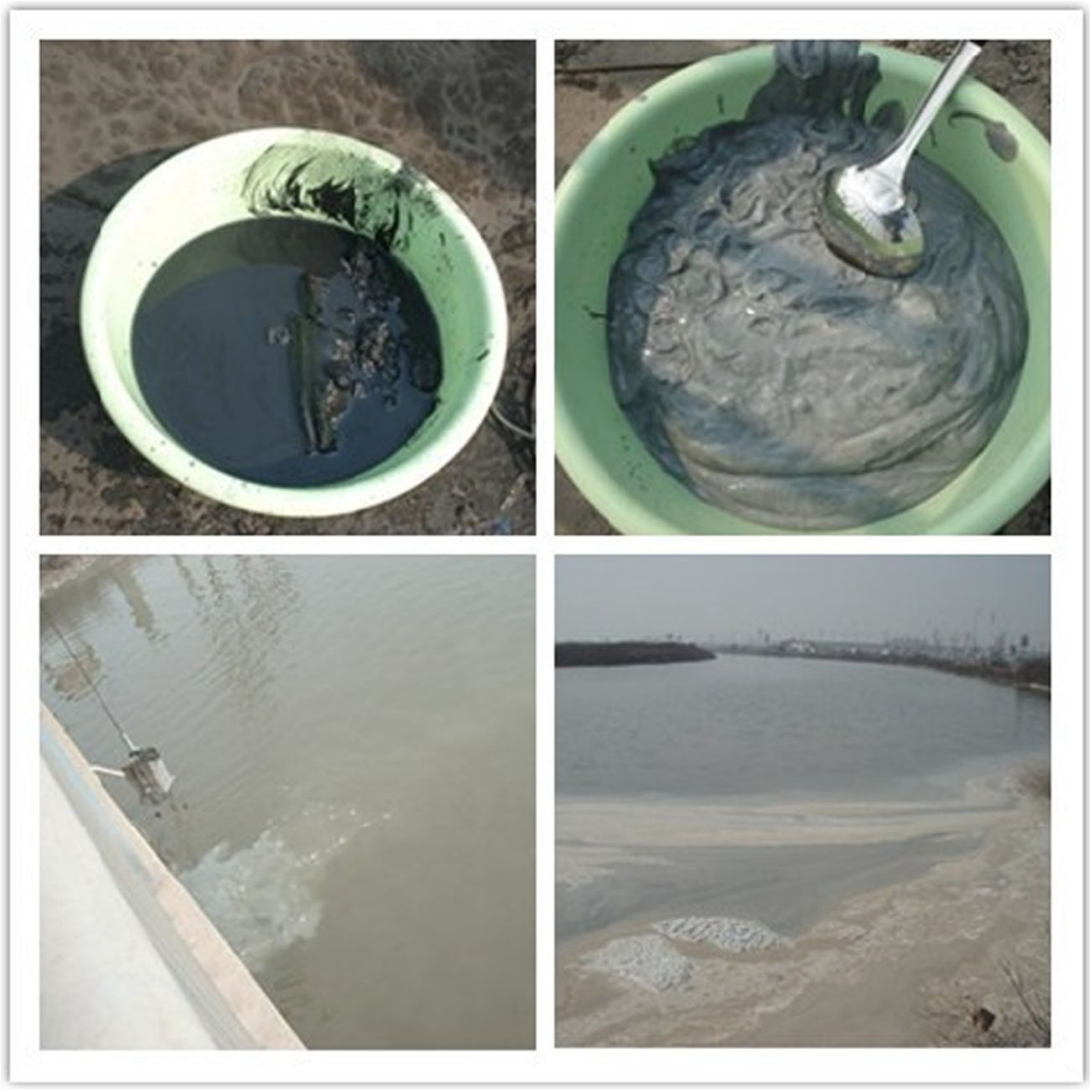
Photographs of sample sites at Dagu Drainage River, Tianjin

The activated sludge contaminated by PCBs was collected from Dagu Drainage River which place is the most heavily polluted (Fig. 2). The 2,3,7,8-TCDD-degrading strain was isolated from the activated sludge.

The 2,3,7,8-TCDD standards were purchased from Cerilliant Corporation. The solvent is n-Nonane and the concentration of 2,3,7,8-TCDD is 50.00 ± 0.32 µg/mL. All solvents (hexane, dichloromethane, acetone, toluene) used in this study were HPLC grade. Anhydrous sodium sulfate was used after heating 6–7 hours at 660 ℃ in muffle furnace. Silica gel was used after heating 6 hours at 550 ℃ in a muffle furnace. All the other chemicals used were analytical grade.

The culture (PDA) was used for the enrichment and isolation of 2,3,7,8-TCDD -degrading strain [23]. The 100 mL medium included that: potatoes extract 20 g, glucose 2.0 g, KH_2_PO_4_ 0.3 g, MgSO_4_ · 7H_2_O 0.15 g, and VB_1_ 10 mg. It also contained 0.1 mL trace element solution (FeSO_4_·7H_2_O 0.01 g, MnCl_2_·4H_2_O 0.01 g and ZnSO_4_·7H_2_O 0.01 g dissolved in 10 mL distilled water).

### Isolation and identification of 2,3,7,8-TCDD-degrading strain

Portions of the activated sludge sample were incubated in 250 mL Erlenmeyer flask containing 100 mL PDA in the presence of 100 µg 2,3,7,8-TCDD. It was pre-cultivated in Erlenmeyer flasks for 3 d at 30 ℃ before all the experiments were conducted. Then 1 mL enriched aqueous culture was transferred to another Erlenmeyer flask with 100 mL PDA and the same amount of 2,3,7,8-TCDD for a subsequent enrichment. After 5 consecutive enrichments had been carried out, dilutions of culture in saline water were incubated on PDA agar plates at 30 ℃ for 7 d. After screening time and again, the fungal colonies were picked according to their morphological analysis. Each isolate was then tested for its ability to degrade 2,3,7,8-TCDD in PDA liquid culture. Then the needed fungus strain was obtained. The morphological and biochemical characteristics of the strain were tested according.

to methods reported in the literature [23]. Further identification was performed using 18S rDNA gene sequencing. The partial 18S rDNA gene sequence was compared to known sequences found in the GenBank database by using Blast similarity searches [24], and closely related sequences were obtained from GenBank.

### Batch experiments

Response surface methodology (RSM) based on the central composite rotatable design (CCRD) was explored to optimize the critical factors and their interactions which significantly affect the 2,3,7,8-TCDD degradation activity of strain QI-1. The significant factors that were selected for independent variables were pH, temperature and inoculum size based on the results of preliminary one-factor-at-a-time experiments [25]. CCRD consisting of 17 experimental runs with three replicates at the center point was generated by design expert [26]. A randomized block design was used and the regression analysis was conducted using the data obtained from the designed experiments.

### Degradation kinetics of 2,3,7,8-TCDD

To determine 2,3,7,8-TCDD degradation kinetics, strain were grown in 100 mL PDA containing 100 µg 2,3,7,8-TCDD in 500 mL flasks and incubated at 30 ℃, with shaking at 200 rpm. Cells were harvest after 72 h and centrifuged at 8000 rpm for 15 min at 4 ℃. The bacterial pellet was washed three times in PDA (PDA without 2,3,7,8-TCDD) and the cells were resuspended in 100 mL PDA with 100 µg 2,3,7,8-TCDD and incubated at 30 ℃ on a rotary shaker (200 rpm). Samples (1 mL) were directly taken from the incubation medium within 144 h and centrifuged at 8000 rpm for 5 min. The supernatant was frozen until analysis by gas chromatography–mass spectrometry (GC/MS).

Also, a blank experiment (didn’t add 2,3,7,8-TCDD, other experimental conditions are the same) should be conducted to assess the concentration of 2,3,7,8-TCDD exactly.

### Identification of 2,3,7,8-TCDD metabolic products

The metabolic products of 2,3,7,8-TCDD in cell-free filtrates of strain cultures grown in PDA containing 1 µg/mL of 2,3,7,8-TCDD were identified by GC/MS. The cell-free filtrates were collected at 1, 2, 3, 4, 5 and 6 days, respectively. The pathway of 2,3,7,8-TCDD degradation by strain QI-1 was illuminated.

### Sample pretreatment

1 mL inocula mixed with 5 mL methylene chloride was added into 10 mL centrifuge tube, ultrasound extraction for 15 min, centrifuged (5000 rpm) for 15 min, the supernatant was collected. The process was repeated three times and the supernatant was combined. And then the extract was purified by a multilayer silica gel column. A glass separation column filled with degreasing cotton in the bottom then filled 1 g activated silica gel, 3 g alkaline silica gel, 1 g activated silica gel, 8 g acidic silica gel and 10 g anhydrous sodium sulfate from the bottom up in sequence. The column was eluted with 70 mL hexane. Then the dioxins in the column were eluted with 90 mL of hexane, and collect fractions into the eggplant-shaped flask. Use the rotary evaporator to condense the fraction and volume to 0.5mL.

### Analytical method

Gas chromatography was used in combination with tandem mass spectrometry for the detection of 2,3,7,8-TCDD and its metabolites. Gas chromatography was carried out under the following conditions: GCMS-QP2010, the carrier gas was Helium at 1 ml/min constant flow, the column was J & W DB-Dioxin( 30 m×0.25 mm ×0.25 µm), sample injection volume was 10 µL, the injector temperature was 250 ℃ and transfer line temperature was 100 ℃, the oven temperature was set as that the start temperature is 100 ℃,10 ℃/min to 280 ℃, keep 2 min. The pressure was 132.6 KPa, total flow speed was 1.80 ml/min. Tandem mass spectrometry was carried out under the following fixed conditions: ion Source Temp: 200 ℃, solvent cut time: 10 min, interface Temp: 200 ℃.

## Results and discussion

### Strain isolation and identification

**Fig. 3:**
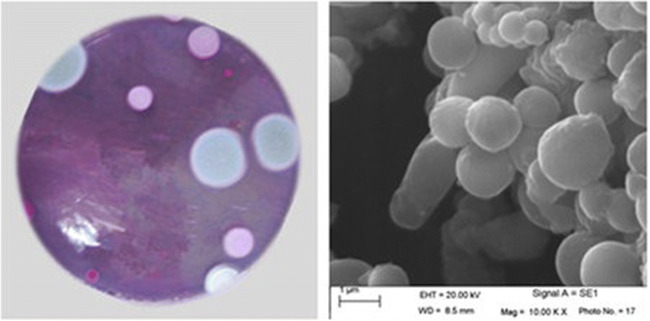
Photographs of a cell of strain QI-1. Colonial morphology photo; SEM photo [10000]

**Fig. 4:**
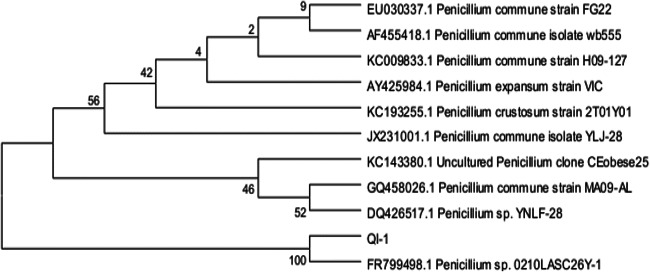
Phylogenetic tree based on 18S rDNA sequences of strain QI-1

As a result of the isolation procedure, seven morphologically different strains were found able to grow well on PDA agar plates containing 1 µg/ mL of 2,3,7,8-TCDD. One of those isolates showing superior degrading ability was selected for further studies. This strain, designated as QI-1, was fungus. From the Fig. 3, the morphological characteristics of colonies of strain QI-1 were the following: 1.5-2 mm in diameter, circular, surface smooth, flat, opaque and round, a little green on the PDA agar plate. Phylogenetic analysis of the 18S rDNA gene sequences revealed that strain QI-1 was grouped among *Penicillium* species and closely clustered with *Penicillium* sp. 0210LASC26Y-1 (GenBank accession number FR799498) with high identities (Fig. 4). Based on the above morphology and 18S rDNA gene analysis, strain QI-1 was identified as *Penicillium* sp.

It was generally considered that the conditions for environmental microorganisms enrichment and screening are crucial in (add the: the selection) selection of desired degrading fungus [26, 27]. In the present study, the screening of 2,3,7,8-TCDD-degrading strain by the method of enrichment procedure from active sludge contaminated with 2,3,7,8-TCDD allowed us to select several fungal isolates which grew well in the presence of 2,3,7,8-TCDD. One most active fungus, showing superior degradation ability was characterized as *Penicillium* sp. Previous studies indicated that the strain from genera *Bacillus* and *Pseudomonas* are metabolically active microorganisms, and they are capable of degrading a variety of aromatic compounds [28–32], while there are hardly any reports on aromatic compounds degrading isolates from *Penicillium* sp. This study provides the first evidence that *Penicillium* species participate in the efficient degradation of 2,3,7,8-TCDD.

The numbers in parentheses represent the sequence accession number in GenBank. The numbers at the nodes indicate bootstrap values. The bar represents sequence divergence.

### Optimization of cultural conditions for 2,3,7,8-TCDD degradation using RSM

RSM was applied to examine the effect of various cultural conditions on enhanced 2,3,7,8-TCDD degradation by *Penicillium* sp. QI-1. 17-run CCRD for three independent variables including temperature (A), pH (B) and inoculum size (C) were manipulated and optimized for enhancement of 2,3,7,8-TCDD degradation (Table 1). The data obtained for percent degradation of 2,3,7,8-TCDD (Y) are representing the combined effect of these three factors at various levels (Table 2). By using RSREG procedure of the Box-Behnken software packages, the following quadratic polynomial model equation (Eq. (1)) was fitted for percent degradation of 2,3,7,8-TCDD in coded process variables.

1 $$\mathrm Y=84.60+3.69\mathrm A+3.73\mathrm B+1.39\;\mathrm C+3.67\mathrm{AB}+0.4925\mathrm{AC}+2.31\mathrm{BC}-10.31\mathrm A^2-8.97\mathrm B^2-0.5232\mathrm C^2$$

- Y—Percent degradation of 2,3,7,8-TCDD.
- A—Coded value of temperature.
- B—Coded value of pH.
- C—Coded value of inoculum size.

**Table 1:** Response surface analysis experimental factor level

Factors	Levels		
	-1	0	1
A Temperature(℃)	15	27.5	40
B pH	4	6.5	9
C Inoculum(g/L)	0.2	0.35	0.5

**Table 2:** Box-Behnken experimental design and results

Run	levels			The degradation of 2,3,7,8-TCDD (%)
	A	B	C	
1	0	0	0	83.42
2	-1	-1	0	62.31
3	0	0	0	85.22
4	0	-1	1	72.34
5	0	0	0	84.13
6	1	0	1	80.64
7	1	-1	0	58.43
8	0	0	0	85.23
9	-1	0	-1	67.88
10	0	0	0	85.02
11	-1	1	0	64.87
12	0	-1	-1	72.87
13	0	1	-1	73.25
14	-1	0	1	68.34
15	0	1	1	81.97
16	1	1	0	75.66
17	1	0	-1	78.21

The reliability of the regression model was analyzed, and the analysis results were shown in Table 3. The regression coefficient R^2^ of the regression equation was 95.93 %, indicating that the regression fitting degree of the model was good, indicating that 95.93 % of the changes in the response value (degradation rate) came from the selected variables, namely, extraction temperature, pH and bacterial liquid concentration. Therefore, the reliability of regression equation is high. The coefficient of variation of Y value C.V.%=3.51, and the lower the value, the higher the credibility of experimental operation. The reliability analysis of the regression model showed that the model could describe the real relationship between the factors and the response value, and the optimal conditions for the degradation of 2,3,7,8-TCDD could be determined by using the regression equation.

**Table 3:** Reliability analysis of regression model

Source	Value
Mean	75.28
R^2^	95.93
Adjust R^2^	90.69
C.V.%	3.51

Analysis of variance (ANOVA) for 2,3,7,8-TCDD degradation by CCRD was presented in Table 4. A p-value < 0.05 suggests that the model is considered to be statistically significant at > 95 % confidence level (Ghevariya et al. 2011). The large value of the regression coefficient (R^2^ = 0.9593) indicates that most of the variation in the response can be explained by the regression model equation. The low coefficient of variation (CV = 3.51 %) also reveals that the model was accurate and reliable. Overall, a regression model for 2,3,7,8-TCDD degradation is significant ( p < 0.01), suggesting that the established quadratic polynomial model for 2,3,7,8-TCDD degradation by strain QI-1 was adequate in representing the actual relationship between response and variables.

**Table 4:** Analysis of variance (ANOVA) for the fitted quadratic polynomial model for 2,3,7,8-TCDD degradation

Source	Sum of Squares	df	Mean Square	F-value	P-value	significance
Model	1152.55	9	128.06	18.32	0.0005	☆☆
A	109.08	1	109.08	15.61	0.0055	☆☆
B	111.01	1	111.01	15.88	0.0053	☆☆
C	15.35	1	15.35	2.20	0.1820	
AB	53.80	1	53.80	7.70	0.0275	☆
AC	0.9702	1	0.9702	0.1388	0.7205	
BC	21.39	1	21.39	3.06	0.1237	
A^2^	447.84	1	447.84	64.07	< 0.0001	☆☆
B^2^	339.03	1	339.03	48.50	0.0002	☆☆
C^2^	1.15	1	1.15	0.1649	0.6968	
Residual	48.93	7	6.99			
Lack of Fit	46.36	3	15.45	24.04	0.0051	
Pure Error	2.57	4	0.6427			
Cor Total	1201.48	16				

*P* value less than 0.05 indicates the model terms are significant (☆); P value less than 0.01 indicates the model terms are extremely significant (☆☆)

**Fig. 5:**
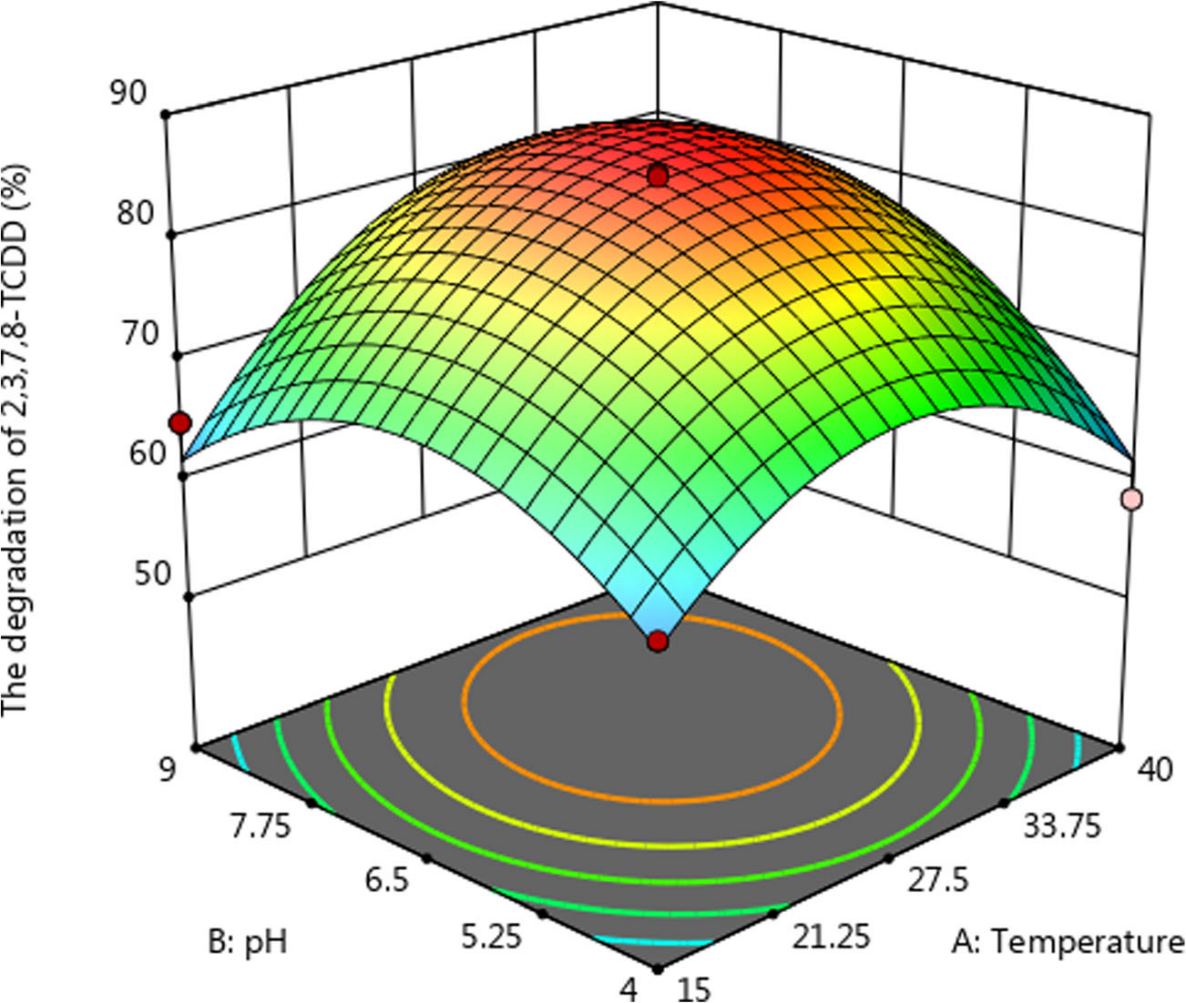
Response surface plot showing the effects of pH and temperature on 2,3,7,8-TCDD degradation by Penicillium sp. QI-1

**Fig. 6:**
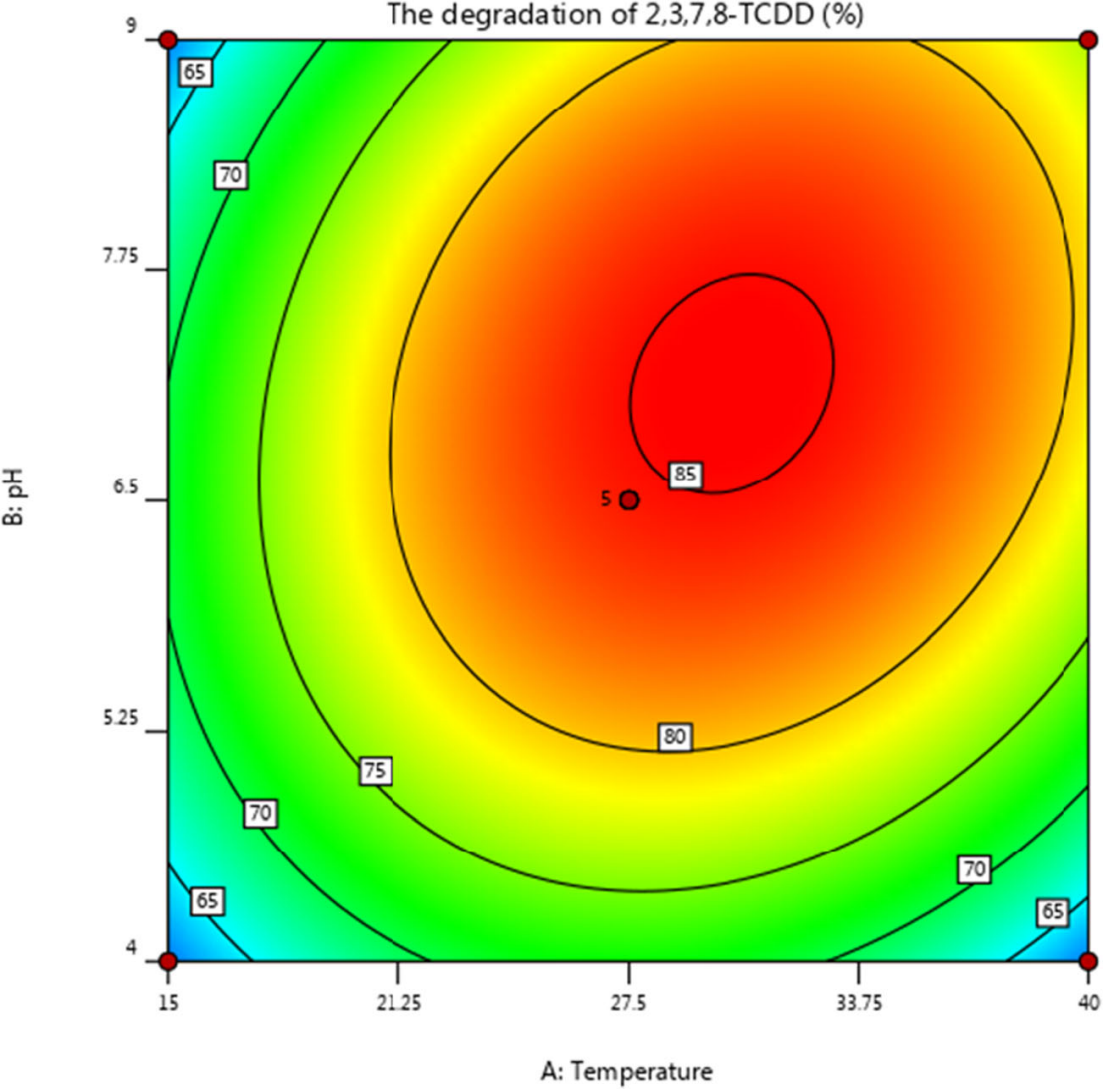
Response surface analysis contour showing the effects of temperature and pH o 2,3,7,8-TCDD degradation by *Penicillium* sp. QI-1

The regression analysis indicates that linear and square terms of temperature (A) and pH (B) played significant roles (p < 0.05) in the degradation of 2,3,7,8-TCDD by strain QI-1, while the linear term of inoculum size (C) and the interaction terms had no significant effects ( p > 0.05) as shown in Table 4. A three-dimensional (3D) response surface was then plotted to illustrate the effects of pH and temperature on 2,3,7,8-TCDD degradation with inoculum size as a constant (Figs. 5 and 6). The model predicts a maximum 2,3,7,8-TCDD degradation of 87.2 % at the stationary point. At the stationary point, the optimum levels for the three variables of temperature (A), pH (B) and inoculum size (C) were observed to be 7.4, 31℃, and 0.5 g/L, respectively. By using the above optimal conditions, the measured degradation rate of 2,3,7,8-TCDD is 87.9 %, which is not much different from the theoretical prediction value. Therefore, the degradation model optimized by response surface analysis method is accurate and reliable, and has practical value.

Previous studies have shown that the application of statistical experimental design techniques in complex processes can result in enhanced yields and allow the rapid and economical determination of the optimum conditions with reduced experiments and minimal resources [33]. RSM is an empirical statistics model that has been successfully applied to improve and optimize biodegradation processes in a variety of microorganisms [32–34]. In the present study, a quadratic polynomial model (Eq. (1)) was successfully developed, and this model could be effectively used for the optimization of 2,3,7,8-TCDD degradation by *Penicillium* strain QI-1.

### Degradation of 2,3,7,8-TCDD

**Fig. 7:**
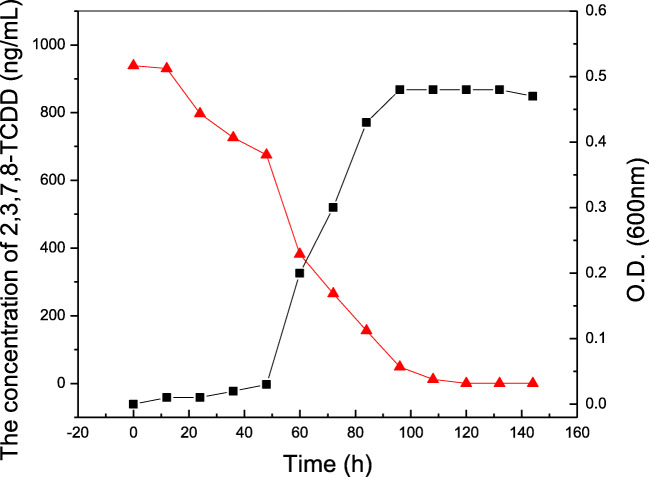
Strain were grown in PDA consisting of 1 µg·mL^− 1^ 2,3,7,8-TCDD for 6 days. Values represent the averages of triplicate determinations. Strain growth (▲); 2,3,7,8-TCDD degradation (■)

When the 2,3,7,8-TCDD-degrading strain was cultivated in the presence of 1 µg/mL 2,3,7,8-TCDD, the concentration of 2,3,7,8-TCDD decreased as shown in Fig. 7. This observation indicates that the decrease in 2,3,7,8-TCDD concentration coincided with the growth of 2,3,7,8-TCDD-degrading strain. The concentration of 2,3,7,8-TCDD decreased during the logarithmic growth phase of 2,3,7,8-TCDD-degrading strain. The concentration of 2,3,7,8-TCDD was determined by GC/MS as shown in Table 5. The highest degrading rate of the strain reached 87.9 % under the optimum conditions.

**Table 5:** The Degradation rate of 2,3,7,8-TCDD by 2,3,7,8--TCDD -degrading strain after 6 days

Number	Bacterial strain	2,3,7,8-TCDD μg/mL		Degradation rate %
		Initial concentration	Finally concentration	
1	Strain	1.000	0.0060	87.9
2	Blank	1.000	0.939	

### Degradation kinetics of 2,3,7,8-TCDD

**Fig. 8:**
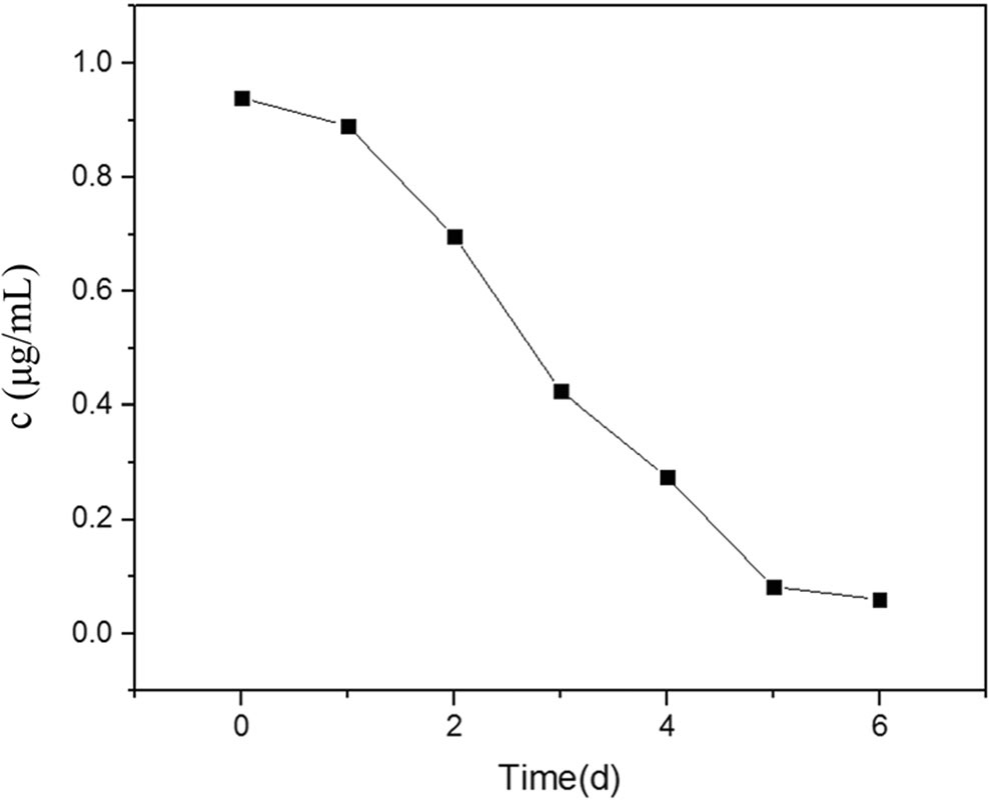
The degradation curve of 2,3,7,8-TCDD during 6days

**Fig. 9:**
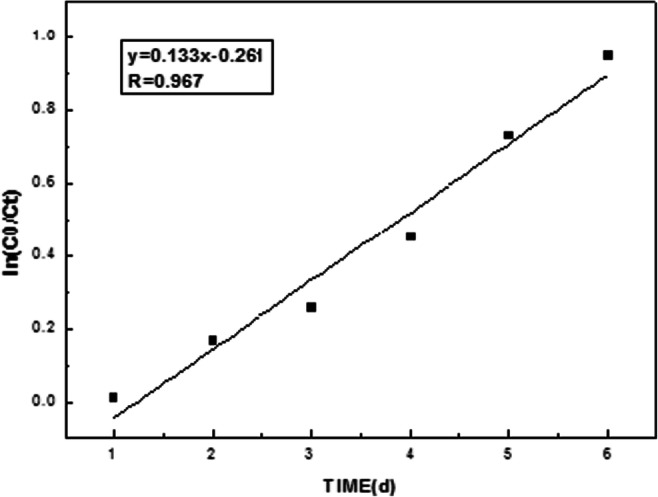
The regression curve between ln(C_0_/C_t_) and t

To confirm the effects on degradation of 2,3,7,8-TCDD by strain QI-1, the biodegradation process was fitted to a first-order kinetic model (Fig. 8). The rate constant ( k) (day^− 1^) was determined using the algorithm *C*_*t*_ = *C*_*0*_ ×e^− k*t*^, where C_0_ was the amount of 2,3,7,8-TCDD at time zero and *C*_*t*_ was the amount of substrate at time *t* (days). Linear regression (ln (*C*_*t*_*/C*_*0*_) of the chemical data and time) was used to calculate the time in which the 2,3,7,8-TCDD concentration in the medium was reduced by 50 % (t_1/2_) [35]. The kinetic equation between ln (*C*_*t*_*/C*_*0*)_ and t was presented in Fig. 9. Kinetic data showed that the degradation process followed the first-order model. According to the kinetic equation ln(*C*_*0*_*/C*_*t*_) = k*t*, the kinetic equation was ln(0.939/C_t_) = 0.133 t, that was C_t_=0.939e^− 0.133t^. Degradation rate constant k = 0.133 d^− 1^ and its half-life was t_1/2_ = ln2/k = 5.21d.

### Identification of the 2,3,7,8-TCDD degradation metabolites

To illuminate the pathway of 2,3,7,8-TCDD degradation by strain QI-1, the metabolic products in cell-free filtrates were extracted and characterized by GC–MS. The GC–MS analysis revealed the presence of five compounds. The five compounds were characterized as 4,5-Dichloro-1,2-benzoquinone, 4,5-Dichlorocatechol, 2-Hydroxy-1,4-benzoquinone, 1,2,4-Trihydroxybenzene and β-ketoadipic acid, respectively, based on the similarity of their fragment and molecular ions with those of corresponding authentic compounds. However, these compounds were transient and they disappeared gradually. At the end of the experiment, no persistent accumulative metabolite was detected by GC–MS, which is agreeable with the findings that strain QI-1 could degrade 2,3,7,8-TCDD.

**Fig. 10:**
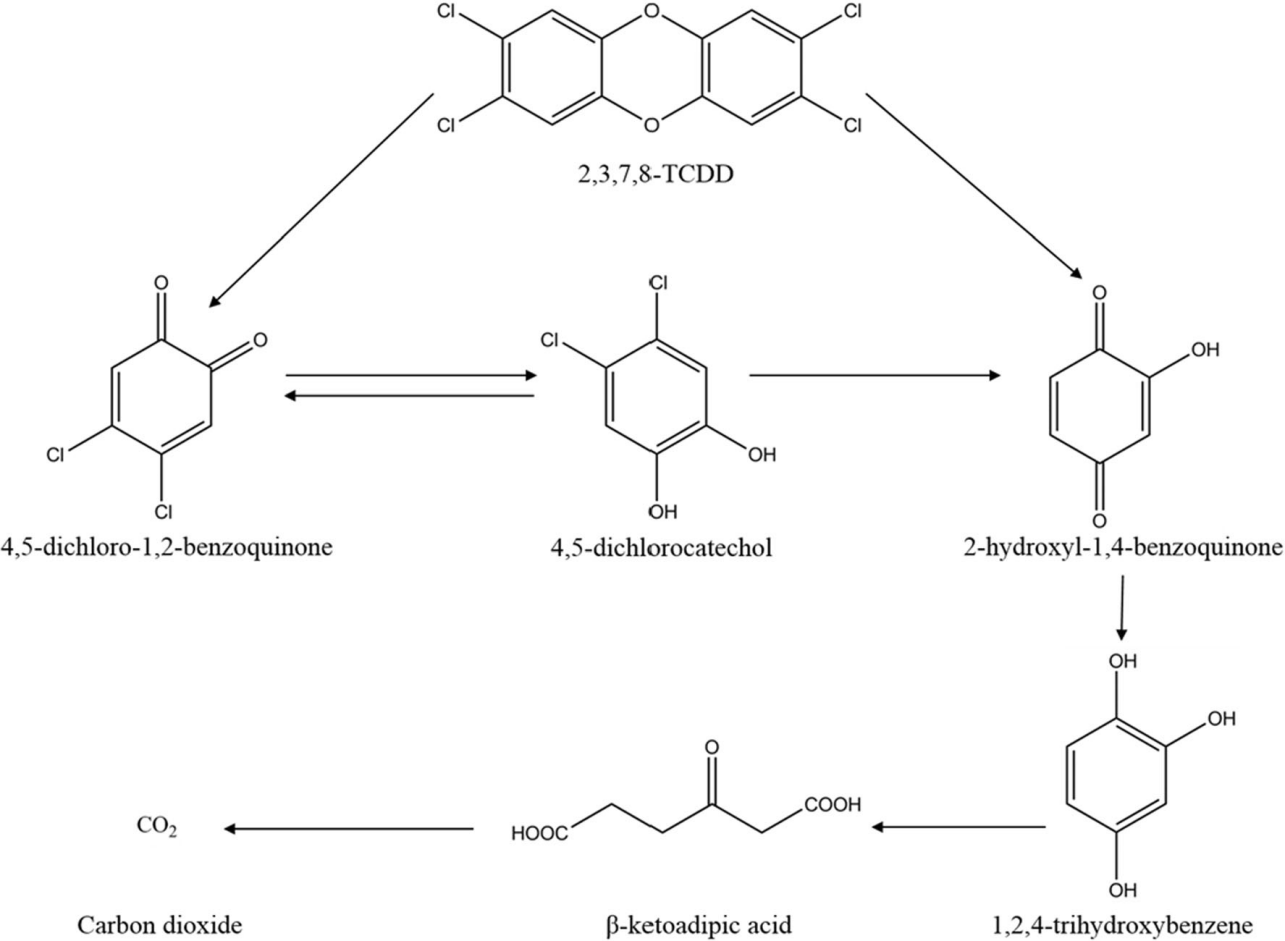
Proposed pathway of 2,3,7,8-TCDD degradation by *Penicillium sp.* QI-1

Based on the metabolites formed, a novel degradation pathway for 2,3,7,8-TCDD in *Penicillium* sp. QI-1 was proposed (Fig. 10). The parent 2,3,7,8-TCDD [1] was first transformed into produce 4,5-Dichloro-1,2-benzoquinone [2] and 2-Hydroxy-1,4-benzoquinone [4]. 4,5-Dichlorocatechol [3] was intermediate. 2-Hydroxy-1,4-benzoquinone [4] then transformed into 1,2,4-Trihydroxybenzene and finally, β-ketoadipic acid [5] was obtained by undergoing aromatic ring cleavage and further metabolism. Therefore, the bacterial strain harbours a complete metabolic pathway for degradation and metabolism of 2,3,7,8-TCDD. This is the first evidence of a novel 2,3,7,8-TCDD detoxification pathway, which we propose is of vital importance in the 2,3,7,8-TCDD biodegradation. The data suggested that the strain QI-1 harboured the metabolic pathway for complete detoxification of 2,3,7,8-TCDD, indicating that the isolate may be ideal for biodegradation of soil and water contaminated with 2,3,7,8-TCDD and related substances.

## Conclusions

Strain QI-1 with the high 2,3,7,8-TCDD degradation ability was isolated from contaminated soil and identified belonging to *Penicillium* sp. It was proved to be promising microorganisms for bioremediation to remove 2,3,7,8-TCDD-containing pollutants from contaminated sites. The optimal pH and temperature for the biodegradation of 2,3,7,8-TCDD by strain QI-1 were pH 7.4 and 31°C. According to the experiment results, it can be calculated that 2,3,7,8-TCDD degradation produced intermediates contained the following five substances: 4,5-dichloro-1,2-benzoquinone, 4,5-dichlorocatechol, 2-hydroxy-1,4-benzoquinone, 1,2,4-trihydroxybenzene and β-ketoadipic acid. β-ketoadipic acid is the key substance for 2,3,7,8-TCDD degradation. It as an intermediate appears in the degradation of 2,3,7,8-TCDD, it is lay a good foundation for further study the degradation mechanism of 2,3,7,8-TCDD. The possibility of using this strain to enhance 2,3,7,8-TCDD removal in a full-scale WWTP will be studied in the future. It may be useful for bioremediation applications. The successful isolation of strain QI-1 provides an opportunity for further research on 2,3,7,8-TCDD – degrading.
